# Duration of sedation effects on the ACTH stimulation test in healthy dogs

**DOI:** 10.1371/journal.pone.0334096

**Published:** 2025-10-09

**Authors:** Jake Salzman, Shelly Olin, Alejandro Esteller-Vico, Luca Giori

**Affiliations:** 1 Department of Small Animal Clinical Sciences, University of Tennessee College of Veterinary Medicine, Knoxville, Tennessee, United States of America; 2 Department of Biomedical and Diagnostic Sciences, University of Tennessee College of Veterinary Medicine, Knoxville, Tennessee, United States of America; Universidade de Trás-os-Montes e Alto Douro: Universidade de Tras-os-Montes e Alto Douro, PORTUGAL

## Abstract

**Background:**

Sedation is frequently required in dogs for diagnostic workups and patient safety. Yet, sedation affects serum cortisol levels; butorphanol elevates cortisol, while dexmedetomidine reduces it dose dependently. Understanding the duration of sedation’s impact on cortisol concentration is important.

**Hypothesis/objectives:**

To evaluate serum cortisol at time 0 and 6-hours post-administration of intravenous (IV) saline (0.5 mL), butorphanol (0.3 mg/kg) and combination of butorphanol and dexmedetomidine (0.3 mg/kg and 5 µg/kg, respectively). Additionally, to compare post-adrenocorticotrophic hormone (ACTH) cortisol concentration at 7 hours after these treatment protocols.

**Animals:**

12 healthy, castrated, colony beagles

**Methods:**

Randomized, controlled, repeated-measure crossover design with a one-week washout between treatments. Serum cortisol was measured at time 0 (T_0_) and 6-hours (T_6_) post-IV administration of saline (0.5 mL), butorphanol (0.3 mg/kg), or combination butorphanol and dexmedetomidine (0.3 mg/kg and 5 µg/kg, respectively). An ACTH-stimulation test was performed at 6-hours post-treatments. Cortisol concentrations were compared with a mixed model analysis with treatment and time as fixed factors.

**Results:**

Mean (± standard deviation) serum cortisol concentration was not significantly different at T_0_ and T_6_ following saline (T_0_ 1.47 ± 1.12; T_6_ 0.91 ± 0.29 µg/dL) butorphanol (0.9 ± 0.41; 0.95 ± 0.38 µg/dL) and combination butorphanol and dexmedetomidine (0.96 ± 0.43; 1.62 ± 0.9 µg/dL) (p = 0.29). There was no significant difference in post-ACTH cortisol for saline, butorphanol, or combination treatment (8 ± 2.25; 8.66 ± 2.26; 8.46 ± 1.83 µg/dL, respectively; p = 0.84)

**Conclusions and clinical importance:**

In healthy dogs following treatment with the aforementioned protocols, cortisol concentration returns to baseline by 6 hours. An ACTH stimulation test started 6-hours post-treatment is not affected by these drug protocols. Additional studies are needed in dogs with adrenal dysfunction.

## Introduction

The ACTH stimulation test is routinely used to assess hypothalamic-pituitary adrenal axis (HPAA) function in dogs [1–3]. The test is recommended for conscious dogs that are not sedated. However, dogs commonly receive sedation during veterinary visits to decrease stress, promote staff safety, and improve diagnostic yield of imaging. In the context of clinical signs and biochemical findings, the results of diagnostic imaging might increase suspicion for adrenal disease. For example, ultrasound findings of an adrenal mass, asymmetrical or abnormally sized adrenal glands, or a biliary mucocele, might prompt further evaluation of adrenal gland function [1,4,5]. A commonly asked clinical question is when HPPA function can be tested following sedation.

Butorphanol is commonly included in sedation protocols for dogs because it produces mild to moderate sedation with minimal cardiopulmonary depression, mild to moderate analgesia, and minimal adverse effects [6]. Butorphanol is a synthetic opioid that acts as a full kappa receptor agonist and a partial antagonist of the mu opioid receptor [6]. Several studies have found that butorphanol at various dosages and routes of administration increases cortisol concentrations in dogs [7–9]. In one study, butorphanol (0.3 mg/kg IV) administered to healthy dogs, 90 minutes prior to testing, significantly increased baseline and post-ACTH cortisol concentrations [7]. In fact, 16% of dogs receiving butorphanol had a post-ACTH cortisol concentration that exceeded a widely used diagnostic cut-off for hyperadrenocorticism (> 20 µg/dL) [7].

Butorphanol is commonly combined with dexmedetomidine, an α-2 adrenoreceptor agonist, for sedation. Use of a combination drug protocol allows for lower dosages of the α-2 adrenoreceptor agonist, a drug that causes peripheral vasoconstriction, marked bradycardia, and decreased cardiac output [10]. There is evidence that dexmedetomidine can decrease cortisol concentrations in a dose-dependent manner [7,8,11]. At dosages commonly used in practice, dexmedetomidine (4 µg/kg) given to healthy dogs IV, 120 minutes prior to testing, did not significantly impact baseline or post-ACTH cortisol [7]. In another study, even a dosage of 10 µg/kg dexmedetomidine IV did not affect baseline cortisol in healthy dogs; post-ACTH cortisol was not assessed [12]. However, a third study demonstrated that baseline and post-ACTH cortisol concentrations were significantly decreased after dogs were administered 80 µg/kg IM or SQ, respectively [11]. No studies have shown the effect of combined dexmedetomidine and butorphanol on baseline cortisol or post-ACTH cortisol.

Additionally, the duration of effect of these sedatives on the HPAA is not known. Dogs administered butorphanol (0.1 mg/kg IM) had increased baseline cortisol concentrations for a minimum of 3–4 hours [8]. In another study of healthy dogs anesthetized with butorphanol (0.4 mg/kg IV) and halothane, baseline cortisol was increased for 5 hours; a change was not seen in dogs treated with halothane alone [9]. Therefore, critical temporal evaluation of these drugs, especially butorphanol, is needed to further elucidate the most appropriate time following their administration to evaluate the HPAA.

The objectives of this study were to 1) compare serum cortisol at time 0 (T_0_) and 6 hours (T_6_) after intravenous (IV) treatment with saline (0.5 mL), butorphanol (0.3 mg/kg), and a combination of butorphanol (0.3 mg/kg) and dexmedetomidine (5 µg/kg) and 2) compare post-ACTH stimulation cortisol at 7 hours (T_7_) for each treatment group. We tested the null hypotheses that serum cortisol concentrations would not be significantly different for dogs in any treatment group at T_0_, T_6_, or T_7_.

## Materials and methods

### Animals

A pilot study was performed on 3 healthy male castrated, adult Beagles. For the main study, there were 12 healthy, adult Beagles, including 7 spayed females and 5 neutered males, from the University of Tennessee research colony. Two dogs from the pilot study were included in the main study. Dogs were included in the study if they had no clinical evidence of hyperadrenocorticism or illness, a normal physical examination on mornings of data collection and a normal CBC and serum biochemical profile within 6 months of data collection. Dogs were excluded if they received any drug in the 30 days prior to the study that could impact the HPAA, including steroids, anti-depressants, anxiolytics and anti-epileptics. All dogs were housed in standard single animal runs throughout the study, and all dogs were part of the colony for a minimum of 6 months before study enrollment. This study was performed, following animal safety guidelines, with approval of The Institutional Animal Care and Use Committee at the University of Tennessee (Protocol number 2942−1222).

### Sample collection and handling

For both the pilot and main study, dogs were fasted a minimum of 12 hours and samples were collected in the morning. On testing days, dogs were removed from the larger housing runs in the morning and temporarily housed in smaller travel kennels. As part of the research colony, all dogs had previous experiences with short-term housing in the travel kennels. For each phlebotomy, dogs were manually restrained with a gentle technique. At each time point, 4 mL of blood was collected with a 20-gauge needle. Blood was drawn preferentially from the jugular vein, but if an insufficient sample was obtained then the saphenous or cephalic veins were utilized. Once the samples from all the dogs were collected for a time point, they were held in a cooled container for 30 minutes to allow clot formation and then centrifuged at 2200 *xg* for 15 minutes. Following centrifugation, serum was transferred into cryovials for storage at −20ºC for 24 hours and then −80ºC pending analysis. After 1 week at −80ºC (pilot study) and 1 week following all sample collection (main study), samples were thawed en masse and analyzed by blinded laboratory personnel.

### Pilot study

Each dog was examined, weighed and baseline cortisol concentration was measured. Dogs were administered 0.3 mg/kg IV butorphanol (Torbugesic®, Zoetis Inc. Kalamazoo, MI). Following drug administration, blood was collected at time points 1.5, 3, 4, 5 and 6 hours to measure cortisol concentration. Twenty-four hours following butorphanol administration an ACTH stimulation test was performed as follows. Blood was collected for pre-ACTH cortisol, dogs were administered cosyntropin (5 µg/kg IV, Cortrosyn^TM^, Amphastar Pharma, Rancho Cucamonga, CA), and 1 hour later the post-ACTH sample was collected.

### Main study design

**Fig 1:**
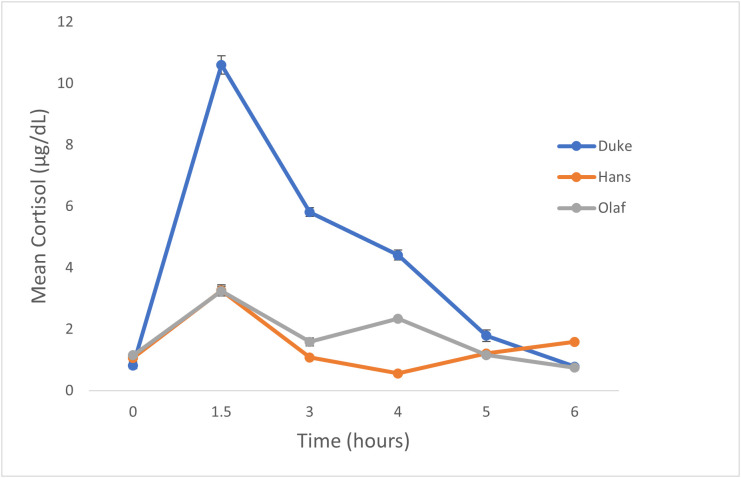
Results of pilot study. Mean (± standard deviation) serum cortisol concentrations of 3 healthy dogs at times 0, 1.5, 3, 4, 5, and 6 hours following intravenous administration of butorphanol (0.3 mg/kg) at time 0. Return to baseline was defined as a cortisol concentration within 30% of the original value.

This was a randomized, controlled, repeated measure study. Dogs were randomized, using a random number generator, into groups of 3 to an assigned day of the week. Each dog was then randomized to treatment, also using random number generation, by week with 1 week between treatments. Treatment groups included saline (0.5 mL IV), butorphanol (0.3 mg/kg IV) and a combination of butorphanol (0.3 mg/kg IV) and dexmedetomidine (5 µg/kg IV, Dexdomitor®, Zoetis Inc. Kalamazoo, MI). Drug dosages were calculated based upon dog weight on the day of the study. Samples were collected at baseline (T_0_), and 6 hours (T_6_) after the assigned treatment. The time point of 6 hours was selected because all dogs (n = 3) in the pilot study had cortisol concentration return to baseline by this time (Fig 1). The return to baseline was defined as a cortisol concentration within 30% of the original value, which accounts for analytical variability [13]. At T_6,_ dogs were given cosyntropin (5 μg/kg, IV) via the cephalic vein. One hour later a post-ACTH blood sample was collected (T_7_).

### Monitoring following drug administration

The degree of sedation was subjective (mild, moderate, severe) and assigned by one investigator [JS] based on response to stimuli and the ability to maintain sternal recumbency.

After drug administration, temperature, heart rate, and respiratory rate were performed every 30 minutes if the dog maintained sternal recumbency. Heart rate and respiratory rate were assessed every 5 minutes if dog was in lateral recumbency. Any dog that had a temperature of less than 98º Fahrenheit (F) was given external heat support until the body temperature exceeded 98º F. Additionally, intramuscular atipamezole (equal volume to dexmedetomidine dosage) could be used at the discretion of the attending clinician for any dog experiencing severe dexmedetomidine sedation effects, defined as apnea, severe bradycardia (heart rate <30 beats/minute), wheels, urticaria or vomiting. Maropitant (1 mg/kg IV; Cerenia^®^, Zoetis Inc. Kalamazoo, MI) was permitted if the dog vomited or was perceived to be nauseated (e.g., lip licking, hard swallowing, ptyalism).

### Hormone analysis

Sample analysis was performed en masse, on thawed samples, following completion of the 3-week study. Cortisol was the only hormone analyzed. Cortisol was measured using a chemiluminescence immunoassay system (Immulite 2000 XPi; Siemens Healthcare Diagnostics Products Ltd., Los Angeles, CA, USA) with a specific cortisol reagent kit (L2KVCO6; Siemens Healthcare Diagnostics Products Ltd., Los Angeles, CA, USA). Quality control was performed in the laboratory daily prior to any sample analysis using K9CON Control 1 and 2 (Siemens Healthcare Diagnostics Ltd.). Each study sample was measured in duplicate, and the average value was recorded. Hormone measurements were performed in accordance with the manufacturer’s instructions by laboratory personnel blinded to dog identity, treatment intervention, and time point.

### Statistical analysis

A priori sample size calculations were performed. The mean (± standard deviation) baseline cortisol from the pilot study was 1.01 ± 0.17 µg/dL. In order to find a 30% increase in cortisol concentration, 4 dogs were needed. Additionally considered was data from 14-healthy dogs showing mean (± SD) post-ACTH cortisol of 12.2 µg/dL (± 3.28) and a within-dog variance of 10.8 µg/dL.^17^ In order to detect a difference >3 µg/dL^17^ in post-ACTH stimulation cortisol concentration with an alpha of 0.05 and a beta of 0.8, then 9 dogs were needed. Therefore, target enrollment was 12 dogs in order to account for dropout.

Descriptive statistics were calculated for each variable and samples analyzed for normality using the Shapiro-Wilk test and for the presence of outliers using box-and-whisker plots. Cortisol concentrations for the 3 treatment groups at T_0_, T_6,_ and T_7_ were compared with a repeated measure mixed model ANOVA as a crossover design, with each dog serving as their own control. Dogs nested within sequence groups were included as a random effect if sequence was significant. Model assumptions regarding equality of variances were verified using Levene’s test for equality of variances. A commercial statistical software package (SAS 9.4 release TS1M5, SAS Institute Inc., Cary NC) was for all analyses. P < 0.05 was considered significant.

## Results

The dogs in the pilot study weighed a median (range) of 14.3 (11.2–16) kg and median (range) age was 3.6 (3–4) years. One male dog was excluded from the main study due to a diagnosis of hypothyroidism 2 months after data collection. Of the remaining 11 dogs included in statistical analysis, the median (range) was 11.4 (10–14.6) kg, median (range) body condition score was 5 (4–6), and median age was 3.6 (3.3–4.25) years.

### Pilot study

Cortisol concentration increased in all dogs by 1.5 hours and returned to baseline within 6 hours (Fig 1).

### Main study: Cortisol concentrations

**Fig 2:**
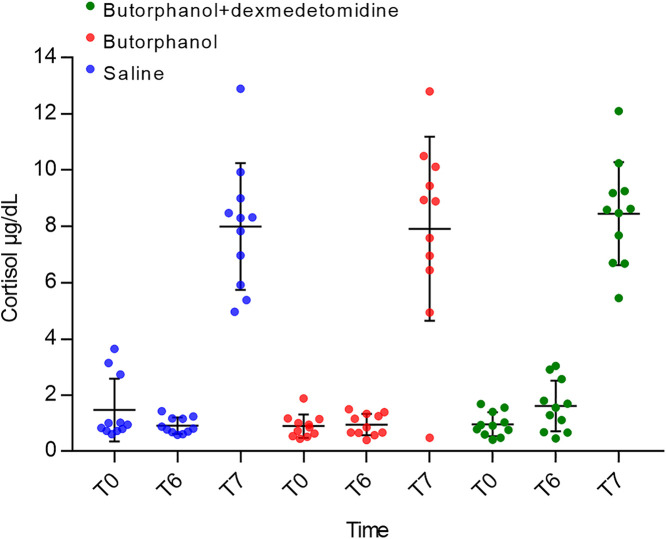
Serum cortisol concentrations at various time points following treatments. Box and whisker plot illustrating the mean serum cortisol concentrations (µg/dL) for 11 healthy Beagles at baseline (T_0_), 6 hours following treatment (T_6_; saline 0.5 mL IV, butorphanol 0.3 mg/kg IV, and combination butorphanol 0.3 mg/kg IV and dexmedetomidine 5 µg/kg IV) and 1 hour post cosyntropin (T_7_; 5 µg/kg IV). There was no statistically significant difference between T_0_ and T_6_ in any treatment group (p = 0.29) or between T_7_ (p = 0.84). Bars represent standard deviation.

Mean serum cortisol concentration was not significantly different at T_0_ and T_6_ following saline, butorphanol, or combination treatment with butorphanol and dexmedetomidine (p = 0.29) (Fig 2). There was no significant difference in cortisol at T_7_ for any treatment group (p = 0.84) (Fig 2).

### Response to sedation

Following the administration of butorphanol alone, all dogs (n = 11, 100%) experienced mild sedation but were able to maintain sternal recumbency. Following administration of butorphanol and dexmedetomidine all dogs experienced moderate sedation, but maintained sternal recumbency by 30 minutes. No dogs required heat support or received atipamezole. All dogs remained normal following saline administration. Nausea and vomiting were not observed in any dog following any treatment and no dogs received maropitant.

## Discussion

In healthy dogs, there is no significant difference in serum cortisol concentration at T_0_ and T_6_ or post-ACTH stimulation cortisol at T_7_ following administration of butorphanol or combination treatment with butorphanol and dexmedetomidine at the dosages used.

There was one outlier dog in the butorphanol group that did not have cortisol stimulation post-ACTH. A definitive cause for this was not established and it is difficult to make conclusions based on one data point. Notably, based on that ACTH-stimulation test, the dog would be diagnosed with hypoadrenocorticism based upon established diagnostic guidelines [3]. However, it is unlikely that the dog truly has hypoadrenocorticism because it had normal stimulation tests with both saline and the butorphanol and dexmedetomidine combination, and had no clinicopathologic changes consistent with hypoadrenocorticism. The possibility of pre-analytical error was considered. An error related to the preparation or administration of cosyntropin (e.g., inadvertent injection of saline) cannot be entirely ruled out. However, there is no known evidence that such an error occurred. It is unlikely that the vial of cosyntropin was reconstituted incorrectly as multiple dogs received cosyntropin from the same vial and no other dog had an abnormal stimulation test. Even if the cosyntropin was inadvertently administered into the perivascular space the dog should still have a normal stimulation test [13]. All samples were handled consistently, making a sample handling error is unlikely. In people, chronic opioid use is associated with adrenal insufficiency through inhibition of ACTH and cortisol production [14,15]. However, this explanation is unlikely to be the case for this study dog because there was no history of chronic opioid use. The outlier stimulation test happened in study week 1 and there was no recent opioid administration. The dog was used for the pilot study, but this preceded the main study by 4 months.

The criterion for cortisol to return to within 30% of baseline in the pilot study was selected to account for the observed or calculated total error (TEo), a combination of systematic and random errors within a laboratory measurement, that has been previously described specifically for the chemiluminescence assay used in our study [13]. The TEo for cortisol on the chemiluminescent assay varies with its concentration. The TEo is generally higher at lower cortisol concentrations and lower at higher cortisol concentrations. Korchia et al. calculated the cortisol TEo for two clinically relevant interpretation thresholds. At the diagnostic threshold of 1.4 µg/dL, TEo is 30%, meaning that for a reported value of 1.4 µg/dL the cortisol could vary within ± 30% or, in other words, between 0.98–1.82 µg/dL. At a higher interpretation threshold of 20 µg/dL, TEo is 20%, so the cortisol could vary within ± 20% or 16–24 µg/dL [13]. Based on a previous cortisol study of dogs in this colony, the TEo for the lower threshold of 1.4 µg/dL was used [7].

This study had several limitations. The most notable is that the study was performed in healthy Beagle dogs. Biologic variability of cortisol within healthy dogs has not been established in the literature, however there is evidence that there are some temporal fluctuations with peak activity in the afternoon [16]. This possible limitation was mitigated at least partially by ensuring all samples were taken at the same time; however, each day with T_6_ around 2 pm and T_7_ around 3 pm near peak cortisol activity. Additional study is needed in other dog breeds and in dogs with disease of the HPAA. This study did not assess cortisol concentrations after sedation administration prior to T_6_ and it is possible that these dogs never had a change in cortisol concentrations following sedation administration. While previous studies [7–9] and the pilot study showed increased cortisol concentration following butorphanol administration, the combination of butorphanol and dexmedetomidine has never been studied and may not result in increased cortisol concentration. Additionally, dogs only received a single dose of sedation. There might be different effects at different drug dosages or with repeated sedation administration.

## Conclusions

In healthy dogs, administered butorphanol (0.3 mg/kg) IV or a combination of butorphanol (0.3 mg/kg) and dexmedetomidine (5 µg/kg) IV, cortisol concentration is not significantly different from baseline at 6 hours. Post-ACTH cortisol is not significantly different when the ACTH-stimulation test is started 6 hours following administration of these drugs. Future studies to assess if these conclusions are consistent in dogs with known adrenal dysfunction or with the use of other sedative medications are recommended to assist in clinical decision making.

## Supporting information

S1 File Raw pilot data. (XLSX)

S2 File Raw study data. (XLSX)
